# Dynamic response and failure characteristics of combined rocks under confining pressure

**DOI:** 10.1038/s41598-022-16299-9

**Published:** 2022-07-16

**Authors:** Cong Ma, Chuanjie Zhu, Jingxuan Zhou, Jie Ren, Qi Yu

**Affiliations:** 1 Faculty of Safety Engineering, China University of Mining and Technology, Xuzhou, 221116 Jiangsu China; 2 Department of Structural Engineering, Tongji University, Shanghai, 200092 China

**Keywords:** Natural hazards, Solid Earth sciences

## Abstract

Gas explosions or coal and gas outbursts can cause transient destruction of combined coal–rock, and the dynamic mechanical response of combined coal–rock masses plays a key role in accident failure, but we now know little about the dynamic mechanical responses of combined coal–rock. In this article, we selected three rocks (limestone, shale, sandstone) and two coals (bituminous coal and anthracite coal) to form combined coal–rock, and analyze their dynamic mechanical properties by using the SHPB system. We find that the dynamic compressive strength and elastic modulus of combined rock–coal are lower than the average value of single rock and coal, while the ultimate strain and strain rate of combined coal–rock are higher than the average values of single rock and coal. Compressive strength and elastic modulus of the combined body increase gradually with increasing confining pressure, and the strain decreases accordingly. The dynamic stress–strain curve demonstrates an obvious double-peak at high strain rate (85.55 s^−1^ and above in the present work), while there is no obvious double-peak of the curve at low strain rate. Dynamic compressive strength of combined coal–rock body increases significantly with increasing confining pressure at low strain rate, but it increases more smoothly at higher strain rate. The elastic modulus also increases with increasing confining pressure, and it seems to be stable as confining pressure increases at low strain rate. The ultimate strain decreases gradually with increasing confining pressure but more gently compared with that at low strain rate. Besides, longitudinal fractures of combined coal–rock bodies include penetrating fractures, partially penetrating fractures, and interrupted fractures stopped at the coal–rock interface. The dynamic mechanical response of combined coal–rock is of guiding significance for maintaining the stability of the roadway and formulating the support measures for the roadway.

## Introduction

**Figure 1:**
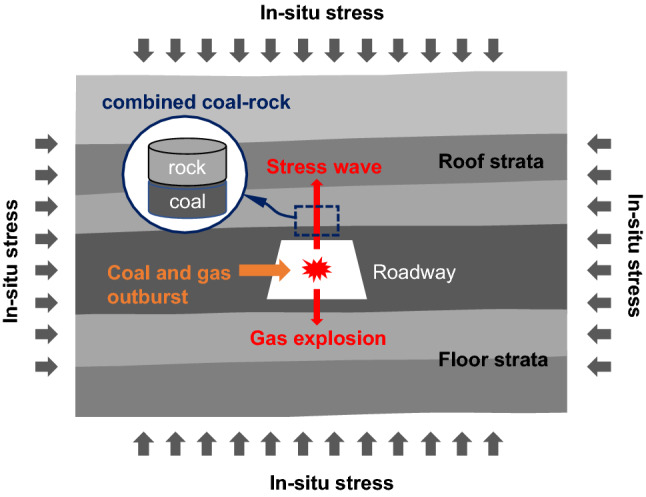
Schematic of dynamic loading impact on combined coal–rock due to gas explosion or coal and gas outburst.

The coal mining industry is threatened by various accidents, such as gas explosions (e.g. premixed methane-air explosion), coal and gas outbursts, and rock bursts^1,2^. These accidents can cause the transient dynamic failure of coal, rock or their combination under high in-situ stress, or even underground roadways collapses eventually, as well as a large number of casualties^3,4^. They occur more frequently with the increase of mining depth. Meanwhile, the mechanical characteristics of coal or rock, which mainly depend on their physical properties, external loading, and other parameters^5^, change greatly due to the increase of mining depth^5,6^. Figure 1 shows accidental dynamic loading caused by gas explosions or coal and gas outbursts. Both gas explosions and coal and gas outbursts directly impact on the coal and surrounding rock, which generates stress wave in coal and strata. Coal reservoirs are typical sedimentary strata composed of different types of rocks. The generated stress wave will propagate across coal and different types of rock. Here we define coal and its contacted rocks in sedimentary strata as combined coal–rock. The dynamic response of combined coal–rock can affect roadway stability under gas explosions or coal and gas outbursts, which is important to mine safety and accident rescue. Additionally, the absence of confining pressure may significantly affect the dynamic response and failure characteristics of combined coal–rock.

Many researchers have investigated dynamic or static mechanical characteristics of coal or rock in the past years^7,8^. As mentioned above, the surrounding rock of underground roadway is not composed of a single layer of coal or rock but a combination of different thickness of surrounding rock and coal (e.g. combined coal–rock). Unfortunately, little is known about the dynamic mechanical characteristics of combined coal–rock. Therefore, the study of mechanical characteristics and failure characteristics of combined coal–rock is of great engineering significance to the explosion-proof design of underground roadways or prevention of coal–rock instability.

Some researchers have studied the quasi-static mechanical properties of combined coal–rock (or so-called coal and rock combined body in some literature). Zuo et al.^9^ found that coal interlayer of weaker strength changes failure mode of coal–rock combined body. Bai et al.^10^ found that the failure of coal and rock in combined bodies is closely related to each other. Chen et al.^11^ found that the mechanical properties and failure of combined bodies are mainly affected by coal. Meanwhile, the mechanical characteristics of combined coal–rock are also affected by some other factors. Zuo et al.^12^ and Guo et al.^13^ studied the mechanical characteristics and failure behavior of coal–rock combined bodies under different confining pressures and different dip angles under static loading, which indicates that triaxial compressive strength of coal–rock combined body increases with increasing confining pressure and decreases with increasing combination dip angle. Wang et al.^14^ studied the mechanical behavior characteristics of coal–rock combination bodies under triaxial conditions and found that the elastic modulus of all specimens increases with increasing confining pressure. Zhang et al.^15^ found that peak strength of coal–rock combined body decreases with increasing height ratio and increases linearly with increasing loading rate.

Coal or rock under dynamic loading behaves quite differently compared with that under static loading. Some researchers have studied the dynamic mechanical properties of coal–rock combination. Yin et al.^16^ studied the effect of temperature on the failure characteristics of coal–rock bodies by Split Hopkinson Pressure Bar (SHPB) testing system and found that degree of fragmentation increases with increasing temperature. Gong et al.^17^ studied dynamic mechanical properties of combined bodies at different loading rates and found that there were two peaks in the stress–strain curve and the loading rate is independent of the first peak and related to the second peak.

Dynamic response of combined rocks is a complicated topic involving many factors, such as rock type, confining pressure, and strain rate which are important for engineering applications. However, previous studies did not reveal the effect of these factors on the dynamic mechanical properties of combined coal–rock. In the present work, we study the dynamic mechanical response of combined coal–rock with different constituent coal and rock.

## Method

### SHPB experimental system

**Figure 2:**
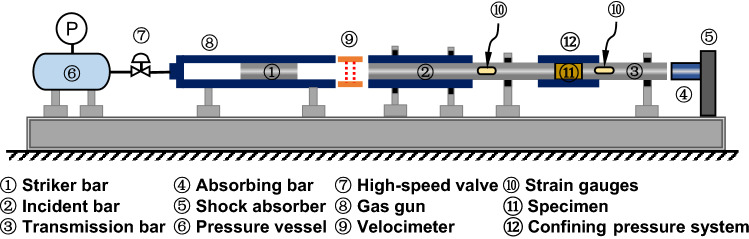
Schematic of general SHPB experimental system.

**Figure 3:**
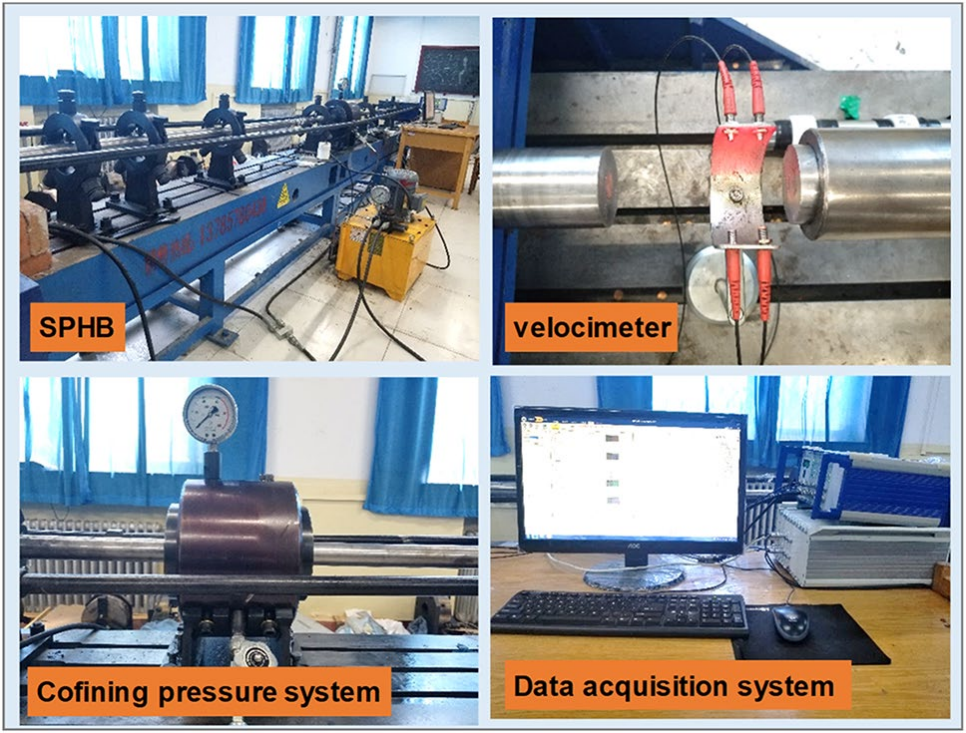
Photograph of the SHPB experimental system.

The dynamic mechanical properties of combined coal–rock are the focus of this paper, and the most reliable and mature test method to obtain the dynamic mechanical properties of combined coal–rock is the SHPB experimental system. And SHPB experimental system has been widely used to test the dynamic mechanical properties of rocks^18^. Schematic and photo of the SHPB experimental system adopted in our experiments are shown in Figs. 2 and 3, which includes a compression bar system, axial compression system, confining pressure system, speed measuring device, and data acquisition system.

The compression bar system includes a striker bar, an incident bar, a transmission bar and an absorption bar. The diameters of these bars are all 50 mm. The length of the striker bar, the incident bar, and the transmission bar is 311.2 mm, 2 m, and 2 m, respectively. The axial and the confining pressure system both include a high-pressure hydraulic pump, a high-pressure oil cylinder, a pressure gauge, and a telescopic shaft pressure bar, respectively. The speed measuring device includes laser gates and a digital oscilloscope. The data acquisition system (DH5960-1, Jiangsu Donghua Test Technology Co., Ltd., China) includes strain gauges, strain gauge bridge box, and dynamic strain amplifier (KD6009, Yangzhou Kedong Electronic Co., Ltd.). In order to ensure the accuracy of the SHPB test, the pulse shaper is pasted at the left end face of the incident bar, and the copper sheet with a diameter of 10 mm and a thickness of 1 mm is used as the pulse shaper.

### Testing principle

SHPB test is based on three basic assumptions^19,20^:

1. Wave propagating in the bars can be described by one-dimensional stress wave propagation theory.
2. Rock or coal is uniformly deformed, and the inertial effect (axial and radial inertial effect) and interface friction effect in the experiment process can be ignored.
3. Rock or coal is under stress equilibrium.

The SHPB test system transforms voltage signal into strain by the equation of Wheatstone bridge^21^. The strain value is calculated by

1 $$\varepsilon \left(t\right)=\frac{2\Delta U(t)}{{\mathrm{K}}_{1}{\mathrm{K}}_{2}{\mathrm{U}}_{0}}$$

where, K_1_ is the sensitivity coefficient of the strain gauge, 2; K_2_ is the gain multiple of the strain gauge, 100; U_0_ is applied bridge voltage, 8 V;$\Delta U(t)$ is the output voltage, V.

Based on one-dimensional stress wave theory^22^, force (*F*_*1*_) at the interface between the coal–rock body and the incident bar and force (*F*_*2*_) at the interface between the coal–rock body and the transmission bar are equal, e.g. *F*_*1*_ = *F*_*2*_, which can be calculated by Zhao and Gary^23^:

2 $${F}_{1}(t)={\mathrm{EA}}_{0}[{\varepsilon }_{i}\left(t\right)+{\varepsilon }_{r}\left(t\right)]$$

3 $${F}_{2}(t)={\mathrm{EA}}_{0}{\varepsilon }_{t}\left(t\right)$$

where, E is Young’s modulus of the bar material, 211 GPa; A_0_ is the cross-sectional area of the bar,1.963 m^2^; ${\varepsilon }_{i}\left(t\right)$, ${\varepsilon }_{r}\left(t\right)$ and ${\varepsilon }_{t}\left(t\right)$ is incident strain, reflection strain and transmission strain, respectively.

Stress $({\sigma }_{1})$ at contact end of the coal–rock body and the incident bar, while stress $({\sigma }_{2})$ at the contact end of the body and transmission bar are^24^:

4 $${\sigma }_{1}(t)=\mathrm{E}\frac{{\mathrm{A}}_{0}}{A}[{\varepsilon }_{i}\left(t\right)+{\varepsilon }_{r}\left(t\right)]$$

5 $${\sigma }_{2}(t)=\mathrm{E}\frac{{\mathrm{A}}_{0}}{\mathrm{A}}{\varepsilon }_{t}\left(t\right)$$

where, A is the cross-sectional area of the coal–rock specimen, 1.963 m^2^.

If stress and deformation of the coal–rock combined body are uniform under dynamic loading, it can be obtained that:

6 $${\varepsilon }_{t}(t)={\varepsilon }_{i}(t)+{\varepsilon }_{r}(t)$$

**Figure 4:**
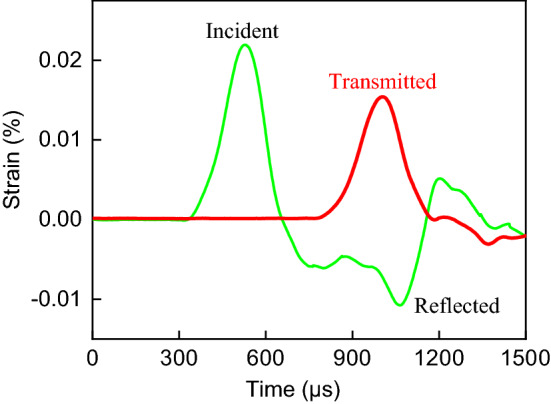
Incident, reflected and transmitted strains.

As shown in Fig. 4, when the striker bar impacts on the incident bar, the longitudinal elastic compression stress wave is generated in the incident bar, and the strain gauge on the incident bar collects the stress wave to obtain the incident pulse ${\varepsilon }_{i}\left(t\right)$. When the stress wave propagates to the interface of the body, part of it is reflected by the incident bar, and the reflected pulse ${\varepsilon }_{r}\left(t\right)$ is collected by the strain gauge. The remainder stress wave passes through the body and propagates in the transmission bar. The transmitted pulse ${\varepsilon }_{t}\left(t\right)$ is collected by the strain gauge on the transmission bar.

The strain rate of coal–rock body $\dot{\varepsilon }(t)$、strain $\varepsilon (t)$ and stress $\sigma (t)$ are expressed by Eqs. (7), (8) and (9) respectively^25^:

7 $$\dot{\upvarepsilon }=\frac{{\mathrm{C}}_{0}}{{\mathrm{l}}_{0}}\left({\upvarepsilon }_{\mathrm{i}}-{\upvarepsilon }_{\mathrm{r}}-{\upvarepsilon }_{\mathrm{t}}\right) \dot{\upvarepsilon }=\frac{{\mathrm{C}}_{0}}{{\mathrm{l}}_{0}}\left({\upvarepsilon }_{\mathrm{i}}-{\upvarepsilon }_{\mathrm{r}}-{\upvarepsilon }_{\mathrm{t}}\right) \dot{\varepsilon }(t)=\frac{c}{{\mathrm{l}}_{0}}[{\varepsilon }_{i}\left(t\right)-{\varepsilon }_{r}\left(t\right)-{\varepsilon }_{t}\left(t\right)]$$

8 $$\varepsilon (t)=\frac{c}{{\mathrm{l}}_{0}}{\int }_{0}^{t}[{\varepsilon }_{i}\left(t\right)-{\varepsilon }_{r}\left(t\right)-{\varepsilon }_{t}\left(t\right)]dt$$

9 $$\sigma (t)=\frac{\mathrm{A}}{2{\mathrm{A}}_{0}}\mathrm{E}[{\varepsilon }_{i}\left(t\right)+{\varepsilon }_{r}\left(t\right)+{\varepsilon }_{t}\left(t\right)]$$

where, l_0_ is the initial length of the body, 0.05 m; *c* is the elastic bar wave speed of the bar material, 5201 m/s.

Substituting formula (6) into formulas (7), (8) and (9), The two-wave method we process data can be concluded that:

10 $$\dot{\varepsilon }(t)=\frac{-2c}{{\mathrm{l}}_{0}}{\varepsilon }_{r}(t)$$

11 $$\varepsilon (t)=\frac{-2c}{{\mathrm{l}}_{0}}{\int }_{0}^{t}{\varepsilon }_{r}(t)dt$$

12 $$\upsigma (t)=\frac{\mathrm{A}}{{\mathrm{A}}_{0}}{E\varepsilon }_{t}(t)$$

### Sample preparation

**Figure 5:**
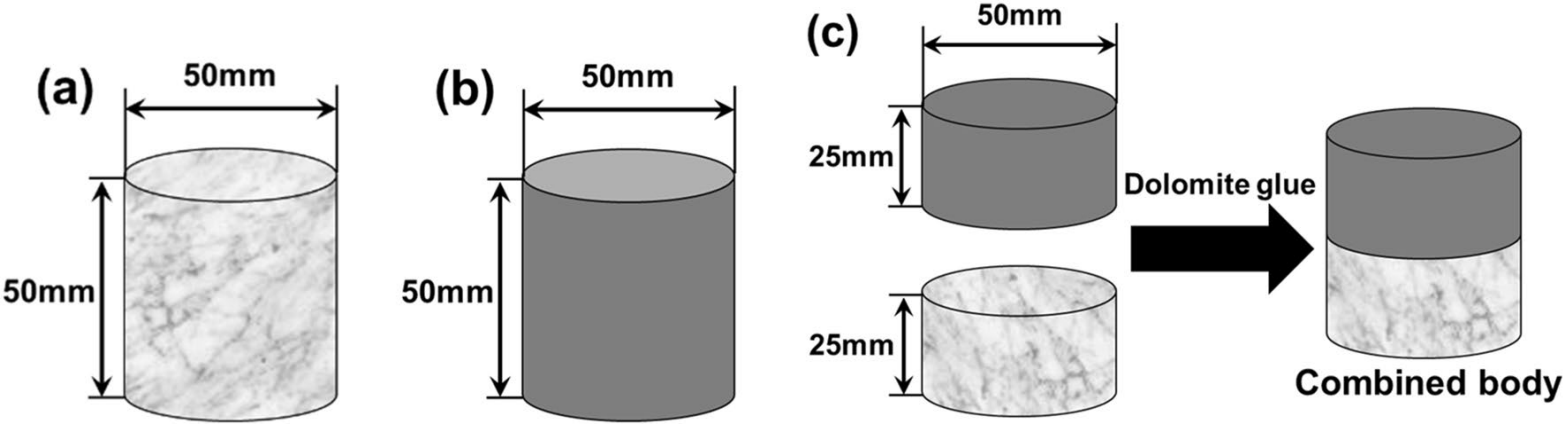
Schematic of testing specimen. (**a**) sandstone, (**b**) bituminous coal, (**c**) combined body.

In our experiments, we used the representative coal and rock commonly found in coal mines, and three typical coal reservoir sedimentary rocks (limestone, sandstone, and shale) and two types of coal (bituminous coal and anthracite) were selected according to the different grades of rock hardness. The basic mechanical parameters of these rocks and coals are shown in Table 1. To minimize the axial and radial inertia effects, the dynamic impact test of SHPB is carried out on a cylindrical body^26,27^. The length of the cylindrical body is 50 mm with a diameter of 50 mm (Fig. 5). The combined coal–rock body is composed of rock and bituminous coal with a length of 25 mm. Because the estimated compressive strength of the combined coal–rock body is much lower than bar material, the sample diameter is the same as the bar diameter to avoid complications from stress concentrations, e.g. 50 mm. Coal and rock of the combined body are bonded by dolomite glue, as shown in Fig. 5c. We used MTS servo press to obtain its static parameters to verify that dolomite glue would not affect the test. Table 2 shows the static parameters of the mean of bonding and splicing specimens (Splicing refers to the direct stacking of two specimens vertically without adhesive). Comparing with spliced specimens, the compressive strength, yield strain and elastic modulus of the bonded specimens were increased by 1.83%, 3.79% and 2.01%, respectively. The error is within 5%, which is within the acceptable range of this experiment. Therefore, the dolomite glue does not significantly change the static mechanical properties of the coal rock mass, and the use of dolomite glue bonded specimens can avoid the experimental errors caused by the slip and contact unevenness of the composite coal–rock samples during the testing process, so that the specimens are more in line with the actual situation of the underground rock layer, so it is reasonable for this experiment to use the dolomite glue.

**Table 1:** Basic mechanical parameters of coal and rock mass.

Sample	Compression strength (MPa)	Elastic modulus (GPa)	Poisson ratio
Anthracite	8.57	6.541	0.201
Bituminous coal	15.96	8.287	0.32
Limestone	48.33	38.37	0.26
Sandstone	20.05	24.68	0.20
Shale	77.98	30.59	0.24

**Table 2:** Mean static parameters of bonded and spliced samples.

Sample	Compression strength (MPa)	Yield strain (%)	Elastic modulus (GPa)
Spliced samples	29.52	1.777	2.585
Bonded samples	28.99	1.712	2.534

During testing, when the combined body is impacted by compressive stress wave, it will generate lateral expansion due to the Poisson effect. If interface lubrication between the body and the bar is insufficient, interface friction will increase, which will introduce experimental error. To reduce the influence of the friction effect on the test results, both ends of the combined body were smeared with lubricant (Vaseline) before tests.

### Ethical approval

This article does not contain any studies with human participants or animals performed by any of the authors.

## Results and discussion

### Stress–strain deformation in combined coal–rock bodies

Bituminous coal, sandstone, and their combined body were selected for the dynamic impact test. Confining and axial pressure is 10 MPa and 5 MPa, respectively. The striking velocity is ~ 6 m/s. Each experiment condition was repeated three times to reduce the contingency of experiments.

Table 3 shows the dynamic test results of coal, rock, and combined bodies. Elastic modulus and dynamic compressive strength of sandstone are the largest under the same conditions, while it is the smallest for bituminous coal. The compressive strength of the combined body is between them. The ultimate strain, strain rate(peak value in test), and peak strain of bituminous coal are the largest, sandstone smallest, and the combined body in the middle.

**Table 3:** Dynamic mechanical parameters of coal, rock, and combined bodies.

Body type	Number	Strain rate (s^−1^)	Compressive strength (MPa)	Ultimate strain (%)	Peak strain (%)	Elastic modulus (GPa)
Sandstone	1	23.43	32.90	0.377	0.231	15.62
	2	27.27	37.93	0.451	0.333	11.80
	3	23.41	32.52	0.423	0.319	9.70
Bituminous coal	1	40.60	14.70	0.669	0.504	5.31
	2	37.34	16.03	0.612	0.463	5.97
	3	44.37	13.51	0.706	0.519	4.87
Combined body	1	36.63	20.90	0.566	0.400	7.53
	2	48.86	23.11	0.653	0.478	6.59
	3	37.03	21.44	0.582	0.441	8.37

**Figure 6:**
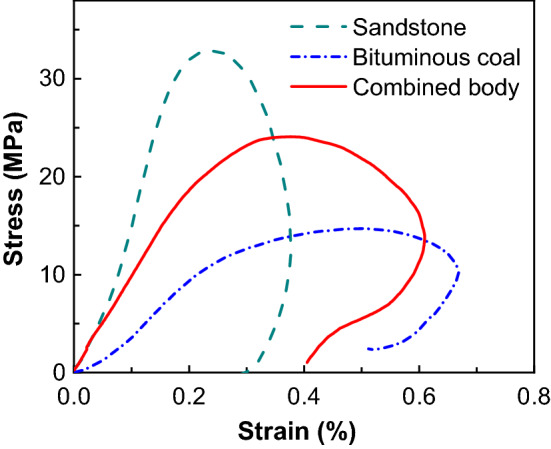
Stress–strain curves of sandstone, bituminous coal and combined bodies.

Additionally, stress–strain deformation in different materials behaves quite differently. Typical stress–strain curves of sandstone, bituminous coal, and combined bituminous coal-sandstone body are given in Fig. 6. Due to the high strength and deformation resistance of sandstone, its ultimate stress is the largest, while its ultimate strain is the lowest. Due to the low strength and porous structure, the strain of bituminous coal changes greatly and the stress changes little. The dynamic mechanical parameters of combined bodies are between bituminous coal and sandstone. This is similar to the results of static tests^28^.

**Figure 7:**
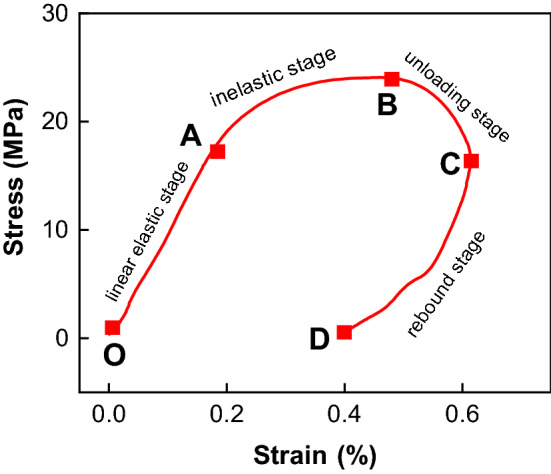
Stress–strain curve of combined bituminous coal-sandstone. OA, linear elastic stage; AB, inelastic stage; BC, unloading stage; CD, rebound stage. Confining pressure = 10 MPa, axial pressure = 5 MPa, striking velocity = 6 m/s.

Taking No.2 combined body given in Table 3 as an example (Fig. 7), the stress–strain curve of the combined body can be divided into four stages, e.g. linear elastic stage (OA), inelastic stage (AB), unloading stage (BC) and rebound stage (CD). Point B is the maximum stress point of the whole curve, its abscissa corresponds to the peak strain. While point C is the maximum strain point of the whole curve, its abscissa corresponds to the ultimate strain.

### Effect of rock type on the dynamic response of combined coal–rock

In our experiments, we select two types of coal (bituminous coal and anthracite) and three types of rocks (sandstone, limestone, and shale) that commonly exist in the sedimentary strata of coal reservoir. These coals and the rock are bonded into six types of combined coal–rock bodies, e.g.

1. combined anthracite-sandstone (AT-SS),
2. combined anthracite-limestone (AT-LS),
3. combined anthracite-shale (AT-SH),
4. combined bituminous coal-sandstone (BT-SS),
5. combined bituminous coal-limestone (BT-LS),
6. and combined bituminous coal-shale (BT-SH).

The confining pressure is set at a constant value (10 MPa), and the axial pressure 5 MPa. The striking velocity is 6 m/s. To avoid randomness of experimental results, we repeat experiments three times for each type of combined coal–rock body.

**Figure 8:**
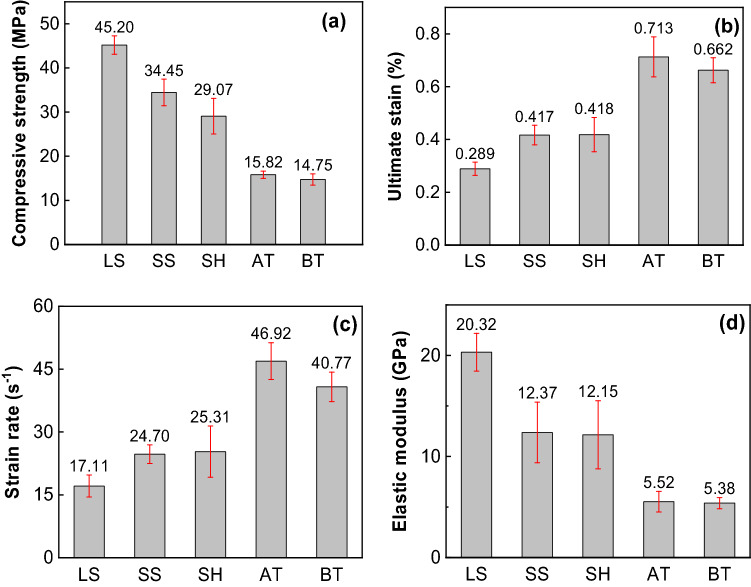
Dynamic mechanical parameters of different types of specimens. (**a**) Compressive strength, (**b**) ultimate strain, (**c**) strain rate, (**d**) elastic modulus.

We tested dynamic mechanical parameters of single rock and coal as background data. Figure 8 gives these parameters of different types of specimens. For the selected rocks and coals, limestone has the highest compressive strength and elastic modulus, followed by sandstone, shale, anthracite, and bituminous coal. The ultimate strain and strain rate of limestone are lowest, which indicates the greatest resistance to deformation due to high compressive strength, while those of anthracite and bituminous coal are higher than rocks.

**Figure 9:**
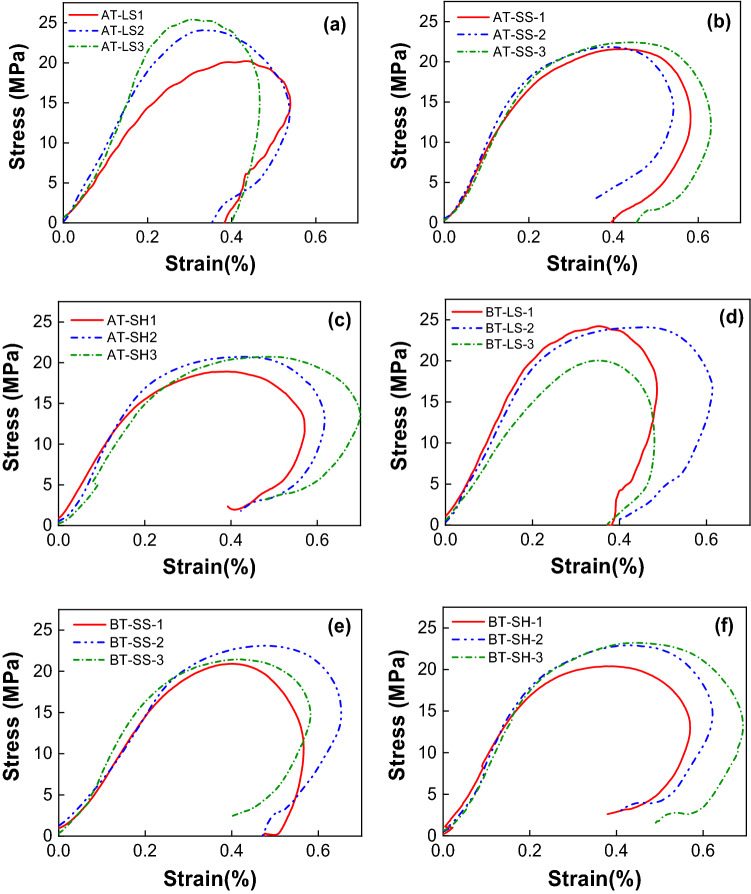
Dynamic stress–strain curves of different types of combined coal–rock bodies. (**a**) Anthracite—limestone, (**b**) anthracite—sandstone, (**c**) anthracite—shale, (**d**) bituminous coal—limestone, (**e**) bituminous coal—sandstone, (**f**) bituminous coal -shale.

**Figure 10:**
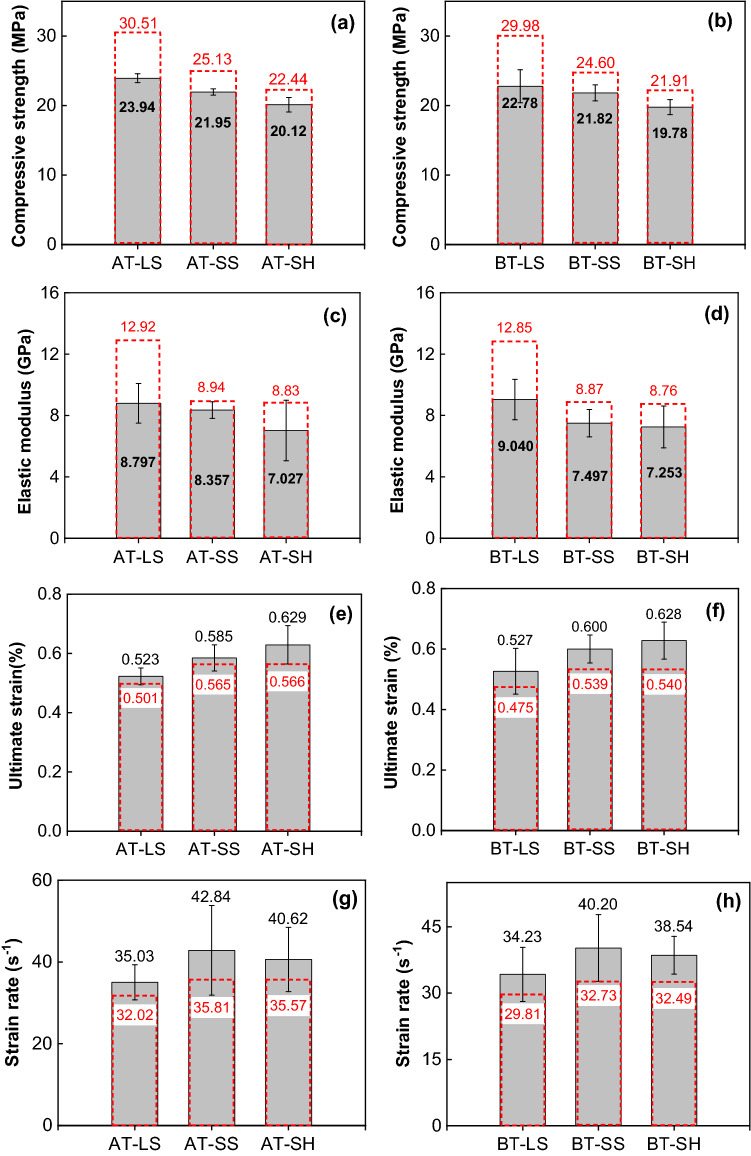
Dynamic mechanical parameters of different types of combined samples. Red dash rectangle represents the average value of single rock and coal, Gray dash rectangle represents the corresponding value of combined samples. *AT-LS* combined anthracite-limestone, *AT-SS* combined anthracite-sandstone, *AT-SH* combined anthracite-shale; *BT-LS* combined bituminous coal-limestone, *BT-SS* combined bituminous coal-sandstone, *BT-SH* combined bituminous coal-shale.

Figure 9 shows the measured dynamic stress–strain curves of different types of combined specimens. Figure 10 gives dynamic mechanical parameters of different types of combined bodies. The average dynamic parameters of corresponding single rock and coal are also given (red dash rectangle in Fig. 10). For example, the compressive strength of anthracite and limestone is 15.82 MPa and 45.2 MPa (see Fig. 8), respectively, so their average value is 30.51 MPa. As mentioned above, the dynamic compressive strength and elastic modulus of single limestone are the highest. As a result, the dynamic compressive strength of the combined anthracite-limestone (AT-LS)) are highest compared with the other two combinations. The same trend is observed for combined bituminous coal–rock. Besides, the dynamic compressive strength of combined anthracite-rock are higher than those of combined bituminous coal–rock. The average value of elastic modulus of the single anthracite and rock is higher than that of the single bituminous coal and rock. The elastic modulus of AT-LS and AT-SH is less than that of BT-LS and BT-SH respectively.

The dynamic compressive strength and elastic modulus of combined rock–coal is lower than the average value of single rock and coal. For example, the compressive strength and elastic modulus of combined anthracite-limestone (AT-LS) is 23.94 MPa and 8.797 GPa, respectively, which is lower than their average values (30.51 MPa and 12.92 GPa, respectively) of single anthracite and limestone. Other combinations have a similar decreasing trend. This indicates that the dynamic compressive strength of coal, which is lower than that of rock in our experiments, has a greater impact on combined coal–rock. Initial failure often occurs in the weakest part of the specimen. The strength of coal is so less than that of rock that the combined coal–rock sample will fail as a whole when the failure of coal occurs. Eventually, the ultimate strain and strain rate follow opposite trends. The ultimate strain and strain rate of combined coal–rock are higher than the average values of single rock and coal, indicating that the anti-deformation ability of combined coal–rock is weak.

### Effect of strain rate on the dynamic response of combined coal–rock

The confining pressure and axial pressure are 10 MPa and 5 MPa, respectively. The specimens are combined bituminous coal-sandstone. The mechanical effects of different dynamic loads on the combined bodies are studied by changing the striking velocity to control the strain rate. Eight different striking velocities were used in our experiments.

**Figure 11:**
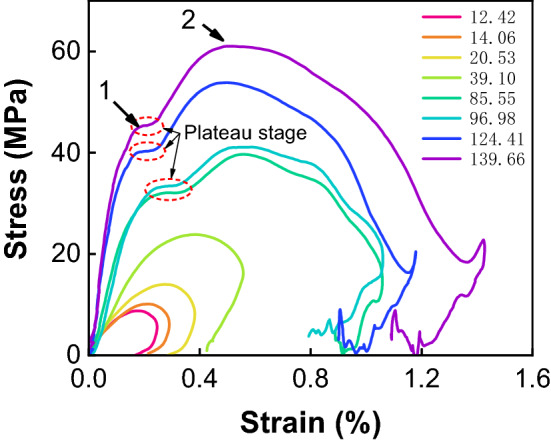
Stress–strain curve of combined bodies.

Table 4 shows dynamic parameters under different strain rates. The dynamic compressive strength, elastic modulus, ultimate strain, and peak strain increase with the increase of strain rate. Figure 11 shows typical stress–strain curves. When the strain rate is less than 85.55 s^−1^, the curve has a smooth dynamic rebound shape. When the strain rate is larger than 85.55 s^−1^, the curve shows a double-peak shape (arrows in the figure.). This also appeared in the study of Gong et al.^17^. In our experiments, there is no obvious double-peak of the curve at low strain rate. However, the curve has a more obvious double-peak and rebound stage with larger vibration at high strain rate. This is the obvious difference in the physical characteristics of combined coal–rock under the action of high strain rate and low strain rate. Besides, there is a plateau stage between the two peaks over a short strain range at high strain rates (85.55 s^−1^ and above in our experiments). The plateau stage usually occurs in cellular or foam material wherein the stress is nearly constant over a large strain range^29^. Because coal and sedimentary rock have many nanoscale micro-pores with limited compact space, the plateau stage is much shorter than that of cellular or foam material. We will conduct a further study on the effect of coal or rock pore distribution on dynamic response.

**Table 4:** Dynamic parameter of combined bodies.

Number	Strain rate (s^−1^)	Compressive strength (MPa)	Ultimate strain (%)	Peak strain (%)	Elastic modulus (GPa)
1	12.42	8.78	0.246	0.180	5.76
2	14.06	10.14	0.290	0.218	6.78
3	20.53	14.00	0.382	0.290	7.40
4	39.10	23.82	0.557	0.386	9.84
5	85.55	39.67	1.058	0.566	21.94
6	96.98	41.10	1.060	0.540	20.98
7	124.41	53.81	1.222	0.499	32.70
8	139.66	61.03	1.297	0.487	38.17

**Figure 12:**
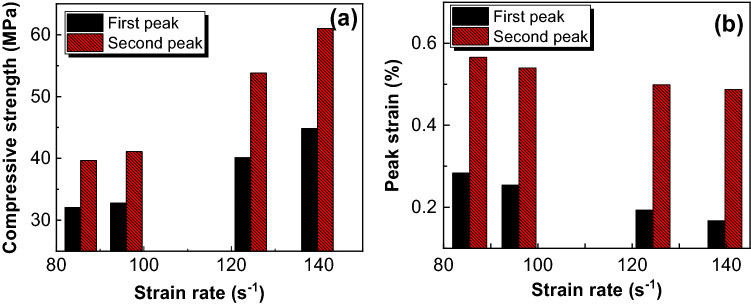
Fitting line of double-peak. (**a**) compressive strength, (**b**) peak strain.

Furthermore, the strain rate has a great influence on the value of double-peaks. Figure 12 presents compressive strengths and peak strains at the first and second peak and their relationships with strain rate. The strain and stress of the second peak are significantly greater than that of the first peak’s. When the strain rate increases, compressive strength’s growth rate of the second peak is greater than that of the first peak, while peak strain’s decrease rate of the first peak is greater than that of the second peak. The compressive strength of the second peak is more affected by the change of strain rate and more sensitive.

Strain rate also has a great influence on the fluctuation of double-peak. It can be seen from Fig. 12 that the differences of stress and strain between the two peaks increase gradually with the increase of strain rate. When dynamic loading increases, the stress compression wave increases, the influence of the combined interface increases, and the stress tensile wave and unloading wave also increase, which leads to the increase of double-peak fluctuation.

**Figure 13:**
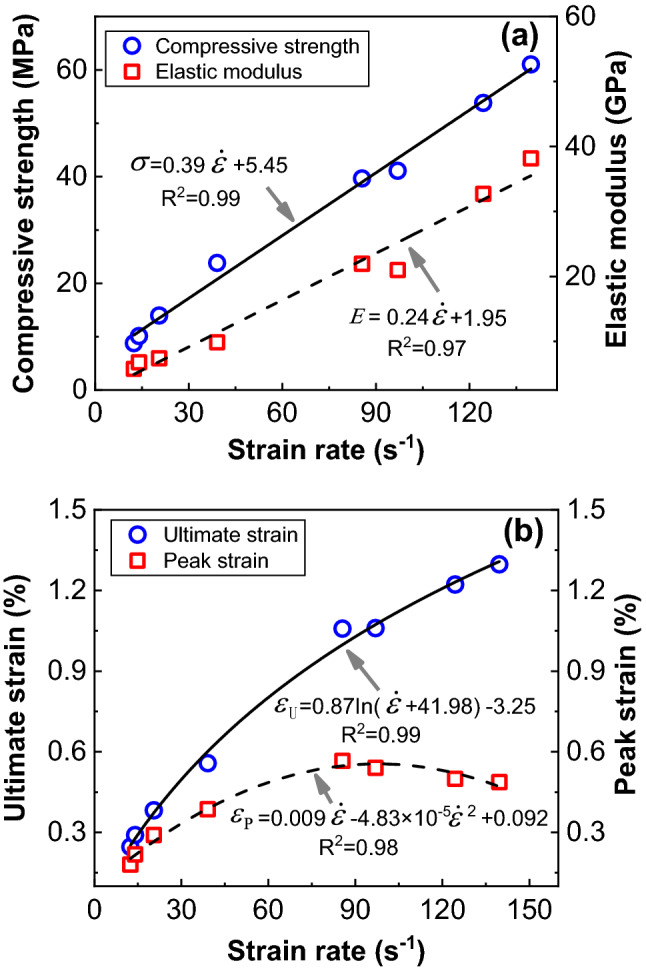
Fitting diagram of dynamic mechanical parameters changing with strain rate. (**a**) compressive strength and elastic modulus, (**b**) ultimate strain and peak strain.

According to the arithmetic mean data in Table 4, the scatter plots of compressive strength, peak strain, ultimate strain, and elastic modulus with the change of strain rate are made and fitted (Fig. 13). The goodness of fit is greater than 0.95. The results show that the relationship between ultimate strain and strain rate is expressed as a logarithmic function, the relationship between peak strain and strain rate is expressed as a quadratic function, and the relationship between elastic modulus, compressive strength and strain rate is expressed as a linear first-order function. With the increase of strain rate, the compressive strength, ultimate strain and ultimate strain increase obviously, and peak strain first increase, then decrease.

### Effect of confining pressure on the dynamic response of combined coal–rock at low/high strain rate

During the experiments, the axial pressure is set as 5 MPa and the striking velocity is ~ 6 m/s. We choose four different confining pressures (0 MPa, 5 MPa, 10 MPa, and 15 MPa) common in coal mining strata. The specimens are combined bituminous coal-sandstone. Each group of tests is also repeated three times.

1. Low stain rate

**Figure 14:**
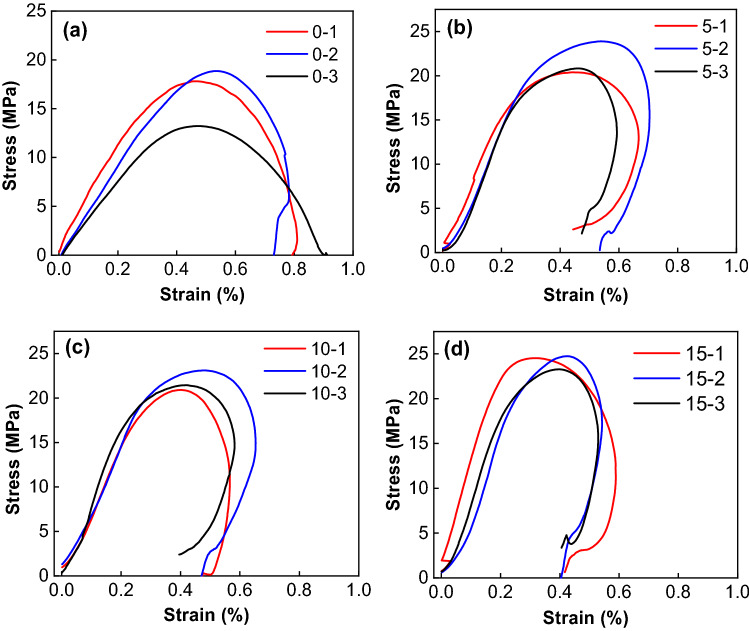
Dynamic stress–strain curves of combined bodies under different confining pressures. (**a**) 0 MPa, (**b**) 5 MPa, (**c**) 10 MPa, (**d**) 15 MPa.

Figure 14 shows dynamic stress–strain curves of combined bodies under different confining pressures at low strain rate. Table 5 gives the dynamic parameters of combined bodies calculated from Fig. 14. As can be seen, the compressive strength and elastic modulus of the combined bodies gradually increase with the increase of confining pressure, and the ultimate strain and strain rate gradually decrease. The pre-existing micro-cracks and pores in the body are compacted under confining pressure. In the process of dynamic impact stress wave propagation, confining pressure restrains the crack growth and improves the plasticity and strength of the combined body^30^.

**Table 5:** Dynamic parameters of combined bodies under different confining pressures.

Confining pressure (MPa)	Number	Strain rate (s^−1^)	Compressive strength (MPa)	Ultimate strain (%)	Elastic modulus (GPa)
0	0-1	41.43	17.82	0.810	5.58
	0-2	38.82	18.90	0.782	4.52
	0-3	45.06	13.25	0.944	4.19
5	5-1	37.84	20.39	0.667	7.21
	5-2	49.12	21.50	0.704	5.01
	5-3	40.40	20.85	0.593	6.34
10	10-1	36.63	20.90	0.566	7.53
	10-2	48.86	23.11	0.653	6.59
	10-3	37.03	21.44	0.582	8.37
15	15-1	52.79	24.57	0.560	9.22
	15-2	33.66	20.33	0.542	6.85
	15-3	36.55	23.34	0.530	9.89

**Figure 15:**
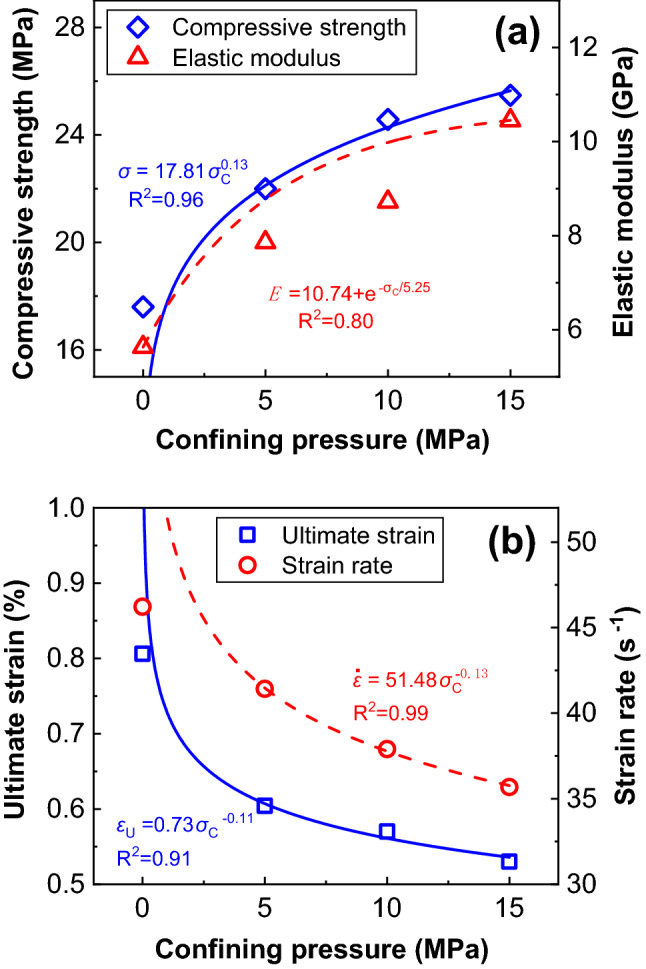
Fitting diagram of mechanical properties of combined bodies under different confining pressures. (**a**) compressive strength and elastic modulus, (**b**) ultimate strain and strain rate.

Hokka^31^ applied confining pressure (0–225 MPa) on rock materials to study the change of the compressive strength. In the present work, the relationship between confining pressure and dynamic compressive strength, ultimate strain, and strain rate are also fitted by a power function. The relationship between confining pressure and elastic modulus is fitted by an exponential function. The R-Squared is above 90% as shown in Fig. 15. The increasing rate of compressive strength decreases with the increase of confining pressure, as well as the reduction rate of ultimate strain and strain rate. Li and Shi^32^ obtained a similar conclusion: the compressive strength of brittle rocks increases with the increase of confining pressure by uniaxial compression experiment and numerical simulation.

**Figure 16:**
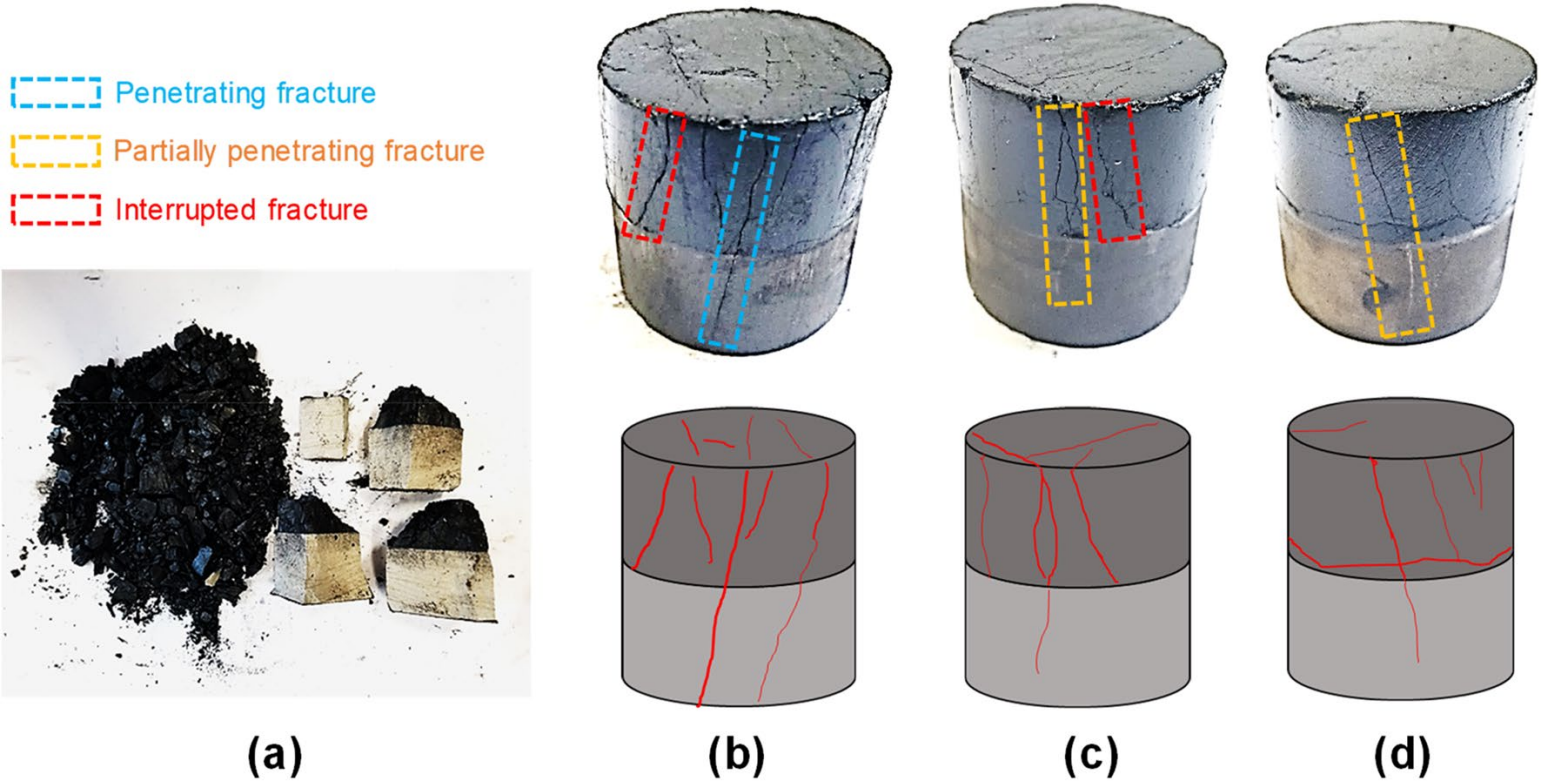
Failure mode and crack propagation in combined samples under different confining pressure. (**a**) unconfined, (**b**) 5 MPa, (**c**) 10 MPa, (**d**) 15 MPa.

Figure 16 gives failure mode and fracture propagation in combined samples under different confining pressure. The unconfined combined coal–rock body broke completely, and the coal was completely pulverized and the rock ruptured into several large blocks. When the confining pressure is 5 MPa, the combined body did not rupture but has many macroscopic fractures, and fractures in coal are much more than that in rock. Macroscopic fractures in combined coal–rock become less when the confining pressure increases to 10 MPa and 15 MPa. Most of macroscopic fractures in the combined body are longitudinal fractures under 5 MPa and 10 MPa confining pressures, which indicates that the confining pressure can inhibit fracture initiation and propagation. When the confining pressure increases to 15 MPa, large transverse fractures appear in the combined body, which means that confining pressure can promote the propagation of transverse fracture. This is similar to the static numerical simulation experiment studied by Chen et al.^30^. With the increase of confining pressure, shear failure along the direction of confining pressure occurs in the combined bodies. In addition, longitudinal fractures include three types:

1. *Penetrating fractures* propagating through coal and rock,
2. *Partially penetrating fractures* propagating through coal and rock but not cracking sufficiently in rock,
3. *Interrupted fractures* stopped at coal–rock interface.

(2) High strain rate.

In the above study, the stress–strain curve of the combined body begins to show double-peak when the strain rate increases to 85.55 s^−1^ and above. It is also found that confining pressure has a significant impact on the dynamic mechanical properties of the combined body. Therefore, the influence of high dynamic impact loading (strain rate greater than 100 s^−1^) under different confining pressure on the mechanics of the combined body was further studied. The mechanical properties of the combined bodies under the confining pressure of 5 MPa, 10 MPa, and 15 MPa at high strain rate were studied.

**Figure 17:**
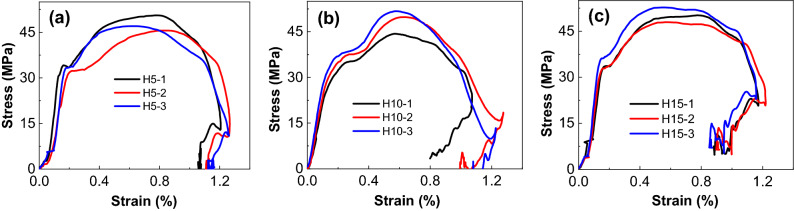
Stress–strain curves of combined bodies with confining pressure under high strain rate. (**a**) 5 MPa, (**b**) 10 MPa, (**c**) 15 MPa.

Dynamic parameters of combined bodies under different confining pressures at high strain rate are shown in Table 6. Figure 17 shows stress–strain curves of combined bodies at high strain rate. Stress–strain curves of the bodies all show double-peak under different confining pressures at high strain rate, indicating that the double-peak is mainly related to the dynamic impact loading. In addition, the stress–strain curve with double-peak appears large vibration at the rebound stage under high strain rate, and the amplitude of vibration increases with the increase of strain rate. The occurrence of vibration is closely related to the confining pressure, which is caused by the incomplete pressure relief of the body under the confining pressure. In other words, the residual strength increases with the increase of confining pressure^33^.

**Table 6:** Dynamic parameters of combined bodies with confining pressure under higher strain rate.

Confining pressure	Number	Strain rate (s^−1^)	Compressive strength (MPa)	Ultimate strain (%)	Peak strain (%)	Elastic modulus (GPa)
5 MPa	H5-1	138.54	50.58	1.206	0.779	26.22
	H5-2	130.78	45.60	1.262	0.643	21.63
	H5-3	134.90	47.01	1.262	0.633	25.37
10 MPa	H10-1	100.04	43.32	1.073	0.565	21.56
	H10-2	135.93	49.83	1.276	0.633	26.31
	H10-3	145.67	51.76	1.338	0.673	27.03
15 MPa	H15-1	113.05	50.17	1.174	0.774	27.29
	H15-2	129.31	47.94	1.220	0.583	31.78
	H15-3	130.83	52.74	1.159	0.554	36.76

**Figure 18:**
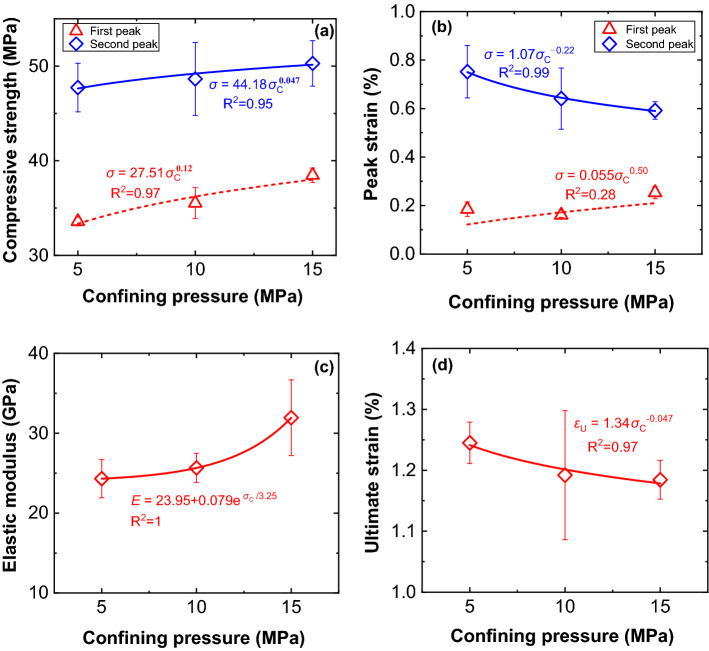
Histogram of mechanical parameters of combined bodies with confining pressure under high strain rate. (**a**) compressive strength, (**b**) peak strain, (**c**) elastic modulus, (**d**) ultimate strain.

Figure 18 shows relationships between dynamic parameters (compressive strength, peak strain, elastic modulus, and ultimate strain) and confining pressures. To compare the dynamic parameter changes at two peaks, compressive strengths and peak strains at two peaks are plotted. Dynamic compressive strength increases significantly with increasing confining pressure at low strain rate (Fig. 15a), but it increases more smoothly at higher strain rate (Fig. 18a), which shows that the confining pressure has no significant effect on the dynamic compressive strength of combined bodies under high dynamic impact loading^31^. Besides, the relationships between compressive strength and confining pressure at two peaks are also fitted well by the power function.

The peak strain decreases gradually with increasing confining pressure for the second peak. The effect of confining pressure on peak strain at the first peak can be fitted well by the power function, but it fits not accurately enough at the first peak. The elastic modulus increases with increasing confining pressure and its relationship with confining pressure are also fitted well by an exponential function. At low strain rate, the elastic modulus seems to be stable when confining pressure increases (Fig. 15a), while it behaves quite differently at high strain rate (Fig. 18c) and indicates a sharper increasing trend when confining pressure increases. Besides, the ultimate strain decreases gradually with increasing confining pressure but more gently compared with that at low strain rate.

## Conclusions

1. The dynamic compressive strength and elastic modulus of combined rock–coal is lower than the average value of single rock and coal, while the ultimate strain and strain rate of combined coal–rock are higher than the average values of single rock and coal.In the above six coal–rock combinations, the AT-LS combination is the optimal combination by comparing compressive strength and elastic modulus.
2. There is no obvious double-peak of the curve at low strain rate. However, the curve has a more obvious double-peak and rebound stage with larger vibration at high strain rate. The relationship between ultimate strain and strain rate is expressed as a logarithmic function, the relationship between peak strain and strain rate is expressed as a quadratic function, and the relationship between elastic modulus, compressive strength and strain rate is expressed as a linear first-order function.
3. Dynamic compressive strength of combined coal–rock body increases significantly with increasing confining pressure at low strain rate, but it increases more smoothly at higher strain rate. The elastic modulus also increases with increasing confining pressure, and it seems to be stable as confining pressure increases at low strain rate, while it indicates a sharper increasing trend when confining pressure increases. The ultimate strain decreases gradually with increasing confining pressure but more gently compared with that at low strain rate.
4. Besides, longitudinal fractures of combined coal–rock bodies include penetrating fractures, partially penetrating fractures, and interrupted fractures stopped at the coal–rock interface.

## Data Availability

The datasets generated during and/or analyzed during the current study are available from the corresponding author on reasonable request.
